# Effect of different training frequencies on maximal strength performance and muscle hypertrophy in trained individuals—a within-subject design

**DOI:** 10.1371/journal.pone.0276154

**Published:** 2022-10-13

**Authors:** Ricardo P. Neves, Felipe C. Vechin, Emerson L. Teixeira, Demostenys D. da Silva, Carlos Ugrinowitsch, Hamilton Roschel, André Y. Aihara, Valmor Tricoli

**Affiliations:** 1 School of Physical Education and Sport, University of São Paulo, São Paulo, SP, Brazil; 2 Diagnostics of the Americas S/A (DASA), São Paulo, SP, Brazil; Universidade Federal de Mato Grosso do Sul, BRAZIL

## Abstract

Several studies comparing resistance training (RT) frequencies may have been affected by the large between-subject variability. This study aimed to compare the changes in lower limbs maximal dynamic strength (1RM) and quadriceps femoris cross-sectional area (CSA) after a RT with different weekly frequencies in strength-trained individuals using a within-subject design. Twenty-four men participated in a 9-week RT program, being randomly divided into two conditions: resistance training with equalized total training volume (**RTEV**) and with unequalized total training volume (**RTUV**). The RT protocol used the unilateral leg press 45° exercise and each subject’s lower limb executed one of the proposed frequencies (one and three times/week). All conditions effectively increased 1RM and CSA (p<0.001); however, no significant differences were observed in the values of 1RM (p = 0.454) and CSA (p = 0.310) between the RT frequencies in the RTEV and RTUV conditions. Therefore, RT performed three times a week showed similar increases in 1RM and CSA to the program performed once a week, regardless of training volume equalization. Nevertheless, when the higher RT frequency allowed the application of a greater TTV (*i*.*e*., RTUV), higher effect size (ES) values (0.51 and 0.63, 1RM and CSA, respectively) were observed for the adaptations.

## Introduction

The main adaptations induced by resistance training (RT) are increased strength and skeletal muscle cross-sectional area (*i*.*e*., muscle hypertrophy). The magnitude of these adaptations can be modulated through the adequate manipulation of some training variables such as intensity, volume, rest interval, and frequency [1, 2]. Among these, training frequency may be defined as the number of weekly sessions [3, 4]. In RT, frequency is usually expressed by the number of times certain muscle groups are exercised in a given period [5], which in turn is influenced by factors such as training intensity and volume, number of muscle groups trained per session, and practitioner training status [6].

Important guidelines on RT prescription [4, 7] based their training frequency recommendations mostly on studies with untrained individuals, hindering the application of their results to individuals with a higher training status. Moreover, in some of these studies, total training volume (TTV, total amount of weight lifted) was not always equalized for comparison of the effects of different RT frequencies, increasing the risk of mistaken conclusions, since TTV can be determinant for increases in muscle strength [8] and mass [9–11].

It should be mentioned that a high TTV in one training session may result in an increased rate of perceived exertion (RPE) [12, 13], greater fatigue accumulation, and slower rate of neuromuscular recovery [14]. On the other hand, increasing RT frequency facilitates TTV distribution over a larger number of sessions, providing favorable conditions for neuromuscular adaptations [15, 16]. Thus, an increase in RT frequency might be an effective strategy to stimulate the neuromuscular system and, consequently, increase strength performance [12, 17, 18].

Regarding muscle hypertrophy, the importance of a higher RT frequency may be related to the maintenance of elevated rates of post-training muscle protein synthesis (MPS). Trained individuals appear to have a shorter period of MPS compared to untrained individuals [19, 20]. Considering that muscle hypertrophy may be affected by accumulated periods of increased post-exercise MPS [21], keeping a higher RT frequency would be an effective strategy to contribute to such adaptation [20, 22]. Furthermore, TTV can be increased with a higher RT frequency [9], and this increase might be related to ribosomal biogenesis [23], satellite cell activation and proliferation, and myonuclear addition which in turn could influence MPS in long term training conditions [24].

Recent reviews and meta-analyzes [2, 25–27] suggest that there is no significant effect of training frequency on either strength performance or muscle hypertrophy when TTV is equalized. However, the results of several studies in these meta-analyzes may have been affected by the large inter-subject variability when different RT frequencies are compared [28]. The comparison of different RT frequencies with between-subject experimental designs and training volume equalization by number of sets and repetitions may not be the most appropriate approach to this problem [29]. Thus, within-subject experimental design should be prioritized [23] if the aim is to evaluate training frequencies with equalized and unequalized TTV. To the best of our knowledge this is the first study, with trained individuals, comparing the effects of RT frequency applying a within-subject experimental design.

Thus, the present study aimed to compare the effects of different weekly RT frequencies (*i*.*e*., one and three times), on maximal strength performance and muscle hypertrophy in trained individuals, using a within- and between- subject experimental designs in two conditions: resistance training with equalized total training volume (**RTEV**) and with unequalized total training volume (**RTUV**). This approach tried to reduce the variability existing in the between-subject designs. We hypothesized that the higher RT frequency would result in larger responses in the dependent variables for both conditions, but in the **RTUV** condition, they would have a greater effect in consequence of a higher TTV over the training period.

## Methods

### Experimental design

The present study followed a longitudinal within and between-subject experimental designs, which investigated the effect of different weekly RT frequencies (*i*.*e*., one and three times) on maximal dynamic strength (1RM) and quadriceps muscle cross-sectional area (CSA) in trained individuals. The participants were classified based on their baseline values of each dependent variable, and counterbalanced divided into two conditions: **RTEV**—resistance training with equalized TTV (*i*.*e*., training volume in the lower limb trained three times being limited by the training volume of the lower limb trained only once) and **RTUV—**resistance training with unequalized TTV (*i*.*e*., training volume in the lower limb trained three times not being limited by the training volume of the lower limb trained only once). All participants performed the unilateral leg press 45° as the RT exercise with each lower limbs submitted to one of the weekly frequencies. The **RTEV** and **RTUV** conditions allowed us to investigate the responses of different RT frequencies in a within-subject experimental design (*i*.*e*., **RTEV** 1x *vs*. **RTEV** 3x and **RTUV** 1x *vs*. **RTUV** 3x) (See Table 1).

**Table 1:** Distribution of subjects’ lower limbs among conditions.

Conditions	Distribution				
**RTEV** (24 lower limbs)	(n = 12 lower limbs) - 1x/week				
				
	(n = 12 lower limbs) - 3x/week				
				
**RTUV** (24 lower limbs)	(n = 12 lower limbs) - 1x/week				
				
	(n = 12 lower limbs) - 3x/week				

RTEV, resistance training with equalized total training volume; RTUV, resistance training with unequalized total training volume. *Note*: In both conditions, the lower limbs were distributed in a balanced way in the different frequencies (*i*.*e*., right lower limbs 1x/week n = 6; left lower limbs 3x/week n = 6; right lower limbs 3x/week n = 6; left lower limbs 1x/week n = 6).

Four weeks before the beginning of the training period, two half-squat 1RM testing sessions were performed (Smith Machine, Hammer Strength®, Rosemont, IL, USA) 72-hours apart. To ensure reproducibility of the test, steps of varying heights (5, 10, and 15 cm) and tapes on the floor were used to determine range of motion (~90° of knee flexion) and feet positioning, respectively. This test was used only to select subjects based on their relative maximum dynamic strength. In the following week, familiarization sessions with the 1RM test in the unilateral leg press 45° were carried on. A minimum of two and a maximum of four sessions (48 hours apart) were necessary to find a reproducible result (variation ≤ 5%) in this test. In the week preceding the beginning of the RT period, all subjects that met the selection criteria performed two evaluation sessions to measure the unilateral leg press 45° 1RM and quadriceps femoris CSA. The same measurements were done one week after the end of the RT program. All participants were instructed not to start any other RT activity for the lower limbs, to maintain their eating habits, and not to use dietary supplements of any category.

### Participants

Twenty-four male participants (26.0 ± 4.0 years; 85.8 ± 15.0 kg and 177.4 ± 6.6 cm), experienced in RT (6.2 ± 4.2 years) were selected for the study. According to the inclusion criteria, all subjects presented a relative maximum dynamic strength (*i*.*e*., 1RM/body mass) in the half-squat exercise ≥ 1.5 (2.1 ± 0.4 kg.kg^-1^). They were free from musculoeskeletal injuries and any other health problems that could prevent them from participating in the study or could affect the results. All participants voluntarily signed, before participation, an informed consent form containing information about the experimental procedures, possible risks and benefits involved. The research project was approved by the by the School of Physical Education and Sport, University of São Paulo Research Ethics Committee (Of.CEP/0317/EEFE/09032017).

### Maximum dynamic strength test (1RM)

The 1RM tests procedures followed the guidelines of the American Society of Exercise Physiology [30]. Participants performed a general warm-up on a treadmill (E750; Movement®, Pompeia, SP, Brazil) running at 9 km/h for five minutes. Then, they performed a specific warm-up with the unilateral leg press 45°, consisting of a set of eight repetitions with an estimated 50% 1RM followed by a set of three repetitions with an estimated 70% 1RM, two minutes apart. The 1RM test started three minutes after the end of the specific warm up. The first leg to be tested was randomly selected, independent of limb dominance. The test measured the maximum amount of weight that could be lifted in a complete movement cycle, which began and ended with the knees in full extension, whereas reaching 90° flexion in the descendent phase of the movement.

The individual adjustments in the leg press 45° equipment (Bolt; Movement®, Pompeia, SP, Brasil) were determined in the first familiarization session. The backrest height was adjusted to individual preference. The foot was comfortably positioned in the middle third of the equipment platform. A goniometer (Shopfisio®, Mogi Guaçu, SP, Brazil) was used to determine the 90° knee range of motion which was marked on the left lateral column of the equipment with the aid of a measuring tape. In order to ensure reproducibility of subsequent tests, all adjustments were recorded in the participants’ assessment sheets. The 1RM value was determined with a maximum of five attempts with three minutes of rest between them.

### Muscle cross-sectional area measurement (CSA)

Quadriceps femoris CSA was obtained by magnetic resonance imaging (Signa LX 9.1; GE Healthcare®, Milwaukee, WI, USA), performed on both lower limbs with the participants positioned supine with their knees extended. An inelastic band was placed on the participants’ feet to contain hip external rotation movement during the measurement. Initially a reference image of the perpendicular distance between the greater trochanter and the lower edge of the lateral epicondyle of the femur was obtained, which was defined as the segment length. Muscle CSA was measured at 50% of the segment length with 0.8 cm scan slice thickness of 3 s duration. The pulse sequence was performed with a field of view between 400 and 420 mm, repetition time of 350 ms, echo time of 9 to 11 ms, two signal acquisitions, and a 256 x 256 mm reconstruction matrix. The CSA was determined using computerized planimetry (Advantage Workstation 4.3; GE Healthcare®, Milwaukee, WI, USA). The image was divided into skeletal muscle, bone and subcutaneous adipose tissue. Next, quadriceps femoris CSA was determined by subtracting bone and subcutaneous adipose tissue area. Images were plotted (OsiriX Lite; Pixmeo Sarl®, Bernex, GE, Switzerland) in duplicate by a specialized blinded researcher, and the mean value between two measurements was used for further analysis.

### Resistance training protocols

The unilateral leg press 45° was the exercise performed during the 9-week RT program. Each participants’ lower limb was submitted to one of the proposed weekly training frequencies (*i*.*e*., one and three times). The participants were divided under two experimental conditions: **RTEV**—resistance training with equalized TTV and, **RTUV—**resistance training with unequalized TTV (Table 1).

Nine exercise sets were performed weekly at both RT frequencies throughout the experimental period, with the number of maximum repetitions changing every three weeks (*i*.*e*., linear periodization model): weeks 1–3 = 12RM; weeks 4–6 = 10RM, and weeks 7–9 = 8RM. All training sessions started with a general warm-up in a treadmill (E720; Movement®, Pompeia, SP, Brazil) running at 9 km/h for five minutes. Next, a specific warm-up composed by 15 repetitions in the unilateral leg press 45° exercise at submaximal intensity (~50%1RM) was done. In the first training session of a week, the lower limb intended for lower RT frequency (once/week) began training by performing nine total sets. Next, the lower limb intended for the higher RT frequency (three times/week) performed only three sets. The other six sets were distributed in the next two RT sessions, separated by a 48-hour interval. In the RTEV condition, repetitions in the higher RT frequency were performed with the average weight (*i*.*e*., TTV/number of repetitions performed) obtained with the lower RT frequency of the contralateral lower limb. A fractional number was often obtained but weight adjustments in sets guaranteed the same TTV for 1x and 3x/week. As an example, the lower limb that trained 1x/week performed a total of 108 repetitions over nine sets, resulting in a TTV of 14,220 kg with an average weight of 131.7 kg/repetition. Therefore, the contralateral lower limb trained with the same TTV executing three sets with 131 kg in day one, and three sets with 132 kg in day two and three with the same number of total repetitions.

### Statistical analysis

Initially the data were analyzed qualitatively and visually for their normal distribution by the Shapiro-Wilk test and the homogeneity of the variances was confirmed by the Levene test. A *one-way* ANOVA was used to test for initial differences in unilateral leg press 45° 1RM and quadriceps femoris CSA between experimental conditions. To evaluate the effect of different weekly RT frequencies on the 1RM and CSA values, a mixed model for repeated measures was used with frequencies (one and three times) and time (pre- and post-training) as fixed factors, and subjects as random factors (SAS 9.3; SAS Institute Inc.®, Cary, NC, USA). Data were reported as mean and standard deviation, with significance level adopted of p≤0.05.

The standard error of measurement (SEM) between two 1RM testing sessions before the training period (*i*.*e*., last familiarization session and pre-training period assessment) and the minimum difference (MD) were calculated based on Weir [31]. Absolute increases above the MD to be considered were reported at the different weekly RT frequencies under the **RTEV** and **RTUV** conditions. For CSA the SEM between two different measurements of the same image was used for calculating the within-researcher reproducibility.

Finally, the effect size (ES) was used to determine the magnitude of the changes, bringing a more practical approach to the obtained results [32, 33]. The ES and confidence interval (CI) were calculated from the mean and standard deviation of Δ at 1RM and CSA values, according to the equation 3 found in Nakagawa and Cuthill [33]. Comparisons were made between the different weekly RT frequencies in the conditions **RTEV** and **RTUV**. The ES was classified as insignificant (<0.19), small (0.20–0.49), medium (0.50–0.79) and large (0.80–1.29) [34]. The upper and lower CI limits that did not cross zero were considered significant.

## Results

Unilateral leg press 45° 1RM and quadriceps femoris CSA baseline values showed no significant differences between **RTEV** and **RTUV** conditions (1RM: F = 0.95536 p = 0.422; CSA: F = 0.01339 p = 0.998) (Table 2).

**Table 2:** Unilateral leg press 45° 1RM and quadriceps femoris CSA values for RTEV and RTUV conditions.

Conditions	Frequency	1RM (kg)		%	Δ (kg)	MD	CSA (cm^2^)		%	Δ (cm^2^)
		pre	post			(13.7 kg)	pre	post		
**RTEV**	1x/week	215.6 ± 53.2	246.8 ± 50.9*	↑ 16.0 ± 10.0	31.2 ± 18.1	↑ 17.5 ± 18.1	100.2 ± 10.6	102.3 ± 11.4*	↑ 2.1 ± 2.1	2.1 ± 2.3
	3x/week	219.0 ± 53.4	252.9 ± 54.3*	↑ 17.2 ± 12.2	33.9 ± 21.6	↑ 20.2 ± 21.6	101.3 ± 10.8	103.3 ± 11.2*	↑ 2.0 ± 2.8	2.0 ± 3.0
**RTUV**	1x/week	186.1 ± 60.7	219.0 ± 65.0*	↑ 19.4 ± 13.1	32.9 ± 19.5	↑ 19.2 ± 19.5	100.4 ± 16.4	101.8 ± 16.4*	↑ 1.5 ± 2.6	1.4 ± 3.0
	3x/week	192.3 ± 65.6	235.9 ± 75.1*	↑ 24.6 ± 14.2	43.7 ± 22.9	↑ 30.0 ± 22.9	100.9 ± 18.7	104.8 ± 17.9*	↑ 4.1 ± 5.0	3.9 ± 4.6

1RM, maximal dynamic strength in the unilateral leg press 45°; Δ, absolute difference; MD, minimum difference; CSA, quadriceps femoris cross-sectional area; RTEV, resistance training

with equalized total training volume; RTUV, resistance training with unequalized total training volume; ↑, increase.

* p<0.001 (time effect).

**Fig 1:**
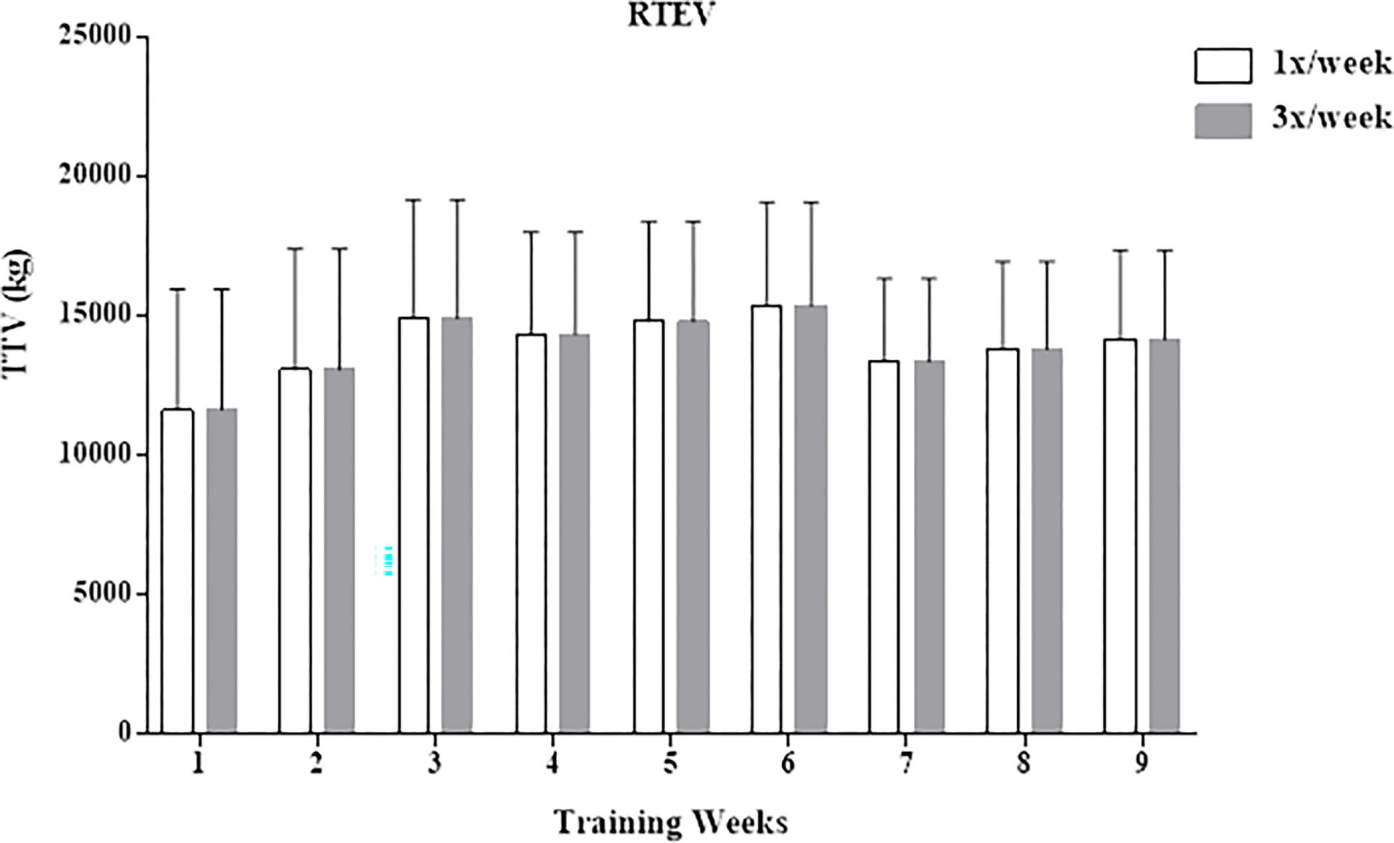
Total training volume per week at different frequencies of RTEV. TTV, total training volume; RTEV, resistance training with equalized total training volume.

**Fig 2:**
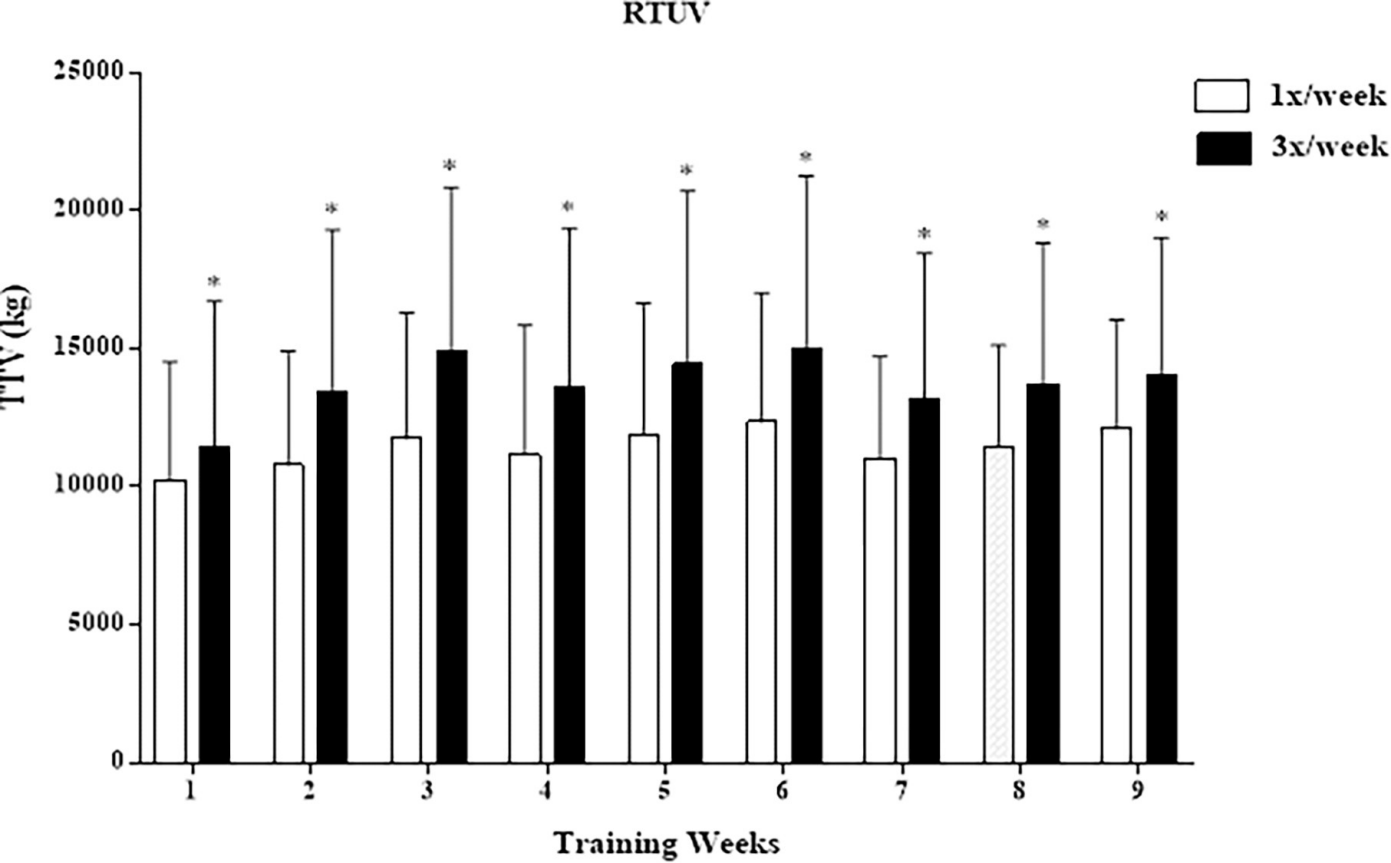
Total training volume per week at different frequencies of RTUV. TTV, total training volume; RTUV, resistance training with unequalized total training volume. *p≤0.05.

There were no significant differences in the average TTV throughout the study between frequencies in the **RTEV** condition (167,582 ± 13,673 kg and 167,586 ± 13,661 kg one and three times/week, respectively, p = 0.999) (Fig 1). On the other hand, in the **RTUV** condition, there were significant differences in TTV between one and three times a week (137,986 ± 8,126 kg and 164,894 ± 12,855 kg, respectively, p = 0.00005) (Fig 2).

In both **RTEV** and **RTUV** conditions, there were significant increases (p<0.001) in the unilateral leg press 45° 1RM after training. However, no significant differences were observed between RT frequencies in each condition (p = 0.454). Similarly, there were significant increases (p<0.001) in the quadriceps femoris CSA values; however, no significant differences (p = 0.310) between RT frequencies were observed (see Table 2). The SEM between 1RM tests was 5.0 kg, which resulted in a MD to be considered of 13.7 kg. The variation between two different measurements of the same image for CSA was 0.2 cm^2^, resulting in a reproducibility of 99.8%.

**Fig 3:**
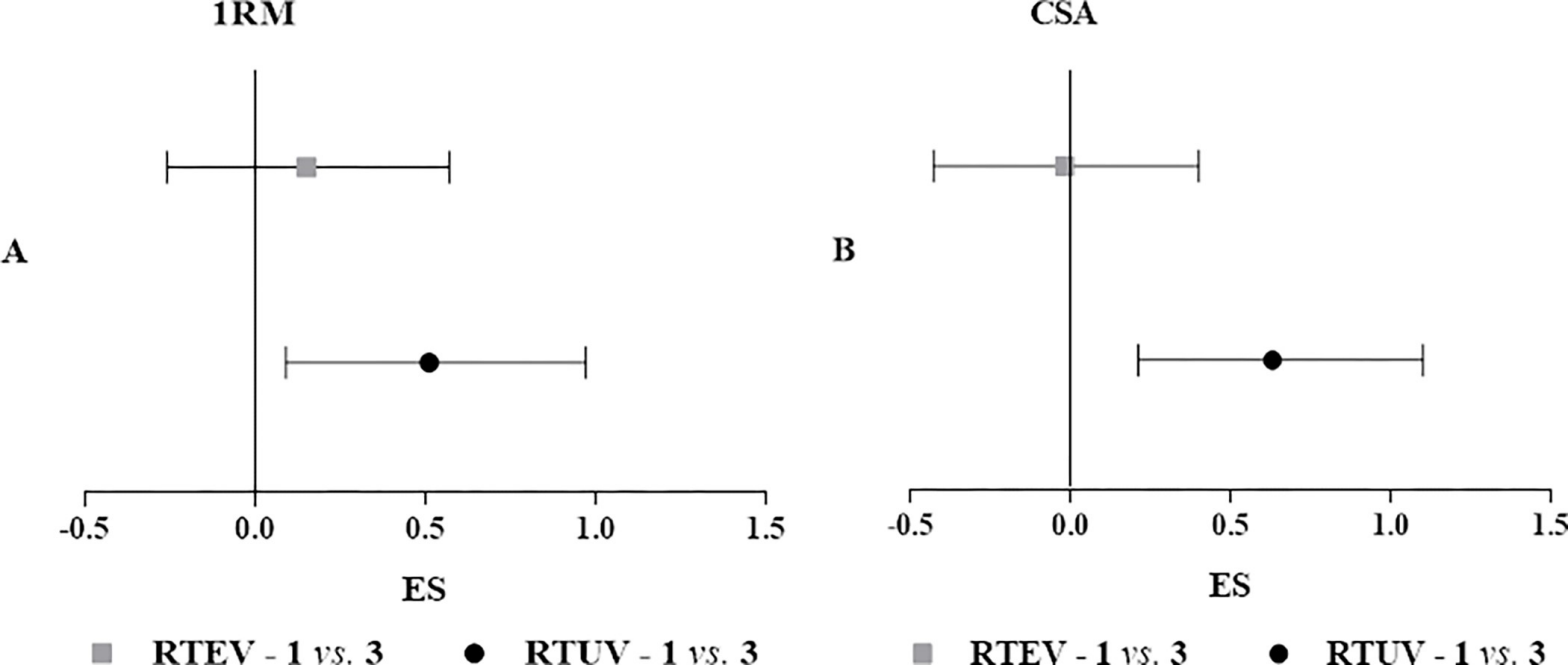
Effect sizes with the confidence interval for 1RM (A) and CSA (B) values at different resistance training frequencies of RTEV and RTUV. ES, effect size; 1RM, maximum dynamic strength; CSA, quadriceps femoris cross-sectional area; RTEV, resistance training with equalized total training volume and; RTUV, resistance training with unequalized total training volume.

The ES was 0.15 (*i*.*e*., insignificant) with a 95% CI from -0.26 to 0.57 between 1RM values of the different frequencies for the **RTEV** condition. On the other hand, in the **RTUV** condition the ES was 0.51 (*i*.*e*., medium) with 95% CI from 0.09 to 0.97 (Fig 3A). For CSA values, the ES between the different frequencies of the **RTEV** condition was -0.02 (*i*.*e*., insignificant) with a 95% CI from -0.43 to 0.40 while in the **RTUV** condition the ES was 0.63 (*i*.*e*., medium) with 95% CI from 0.21 to 1.10 (Fig 3B). Therefore, the higher RT frequency (higher TTV) induced significant increases in the **RTUV** condition for 1RM and CSA, since for both variables the upper and lower CI limits did not crossing zero (Fig 3A and 3B).

**Fig 4:**
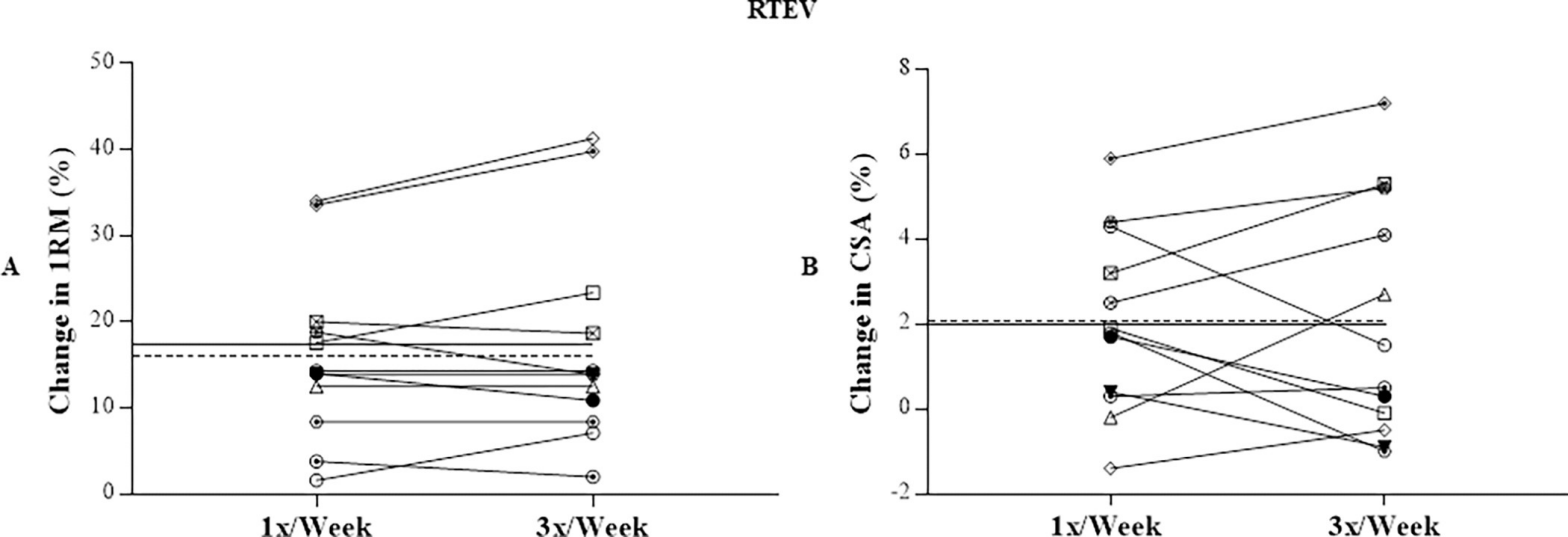
Individual percentage change in the values of 1RM (A) and CSA (B) in the RTEV. 1RM, maximum dynamic strength; CSA, quadriceps femoris cross-sectional area; RTEV, resistance training with equalized total training volume; ———, average of the percentage change 1x/week;–––––, average of the percentage 3x/week.

**Fig 5:**
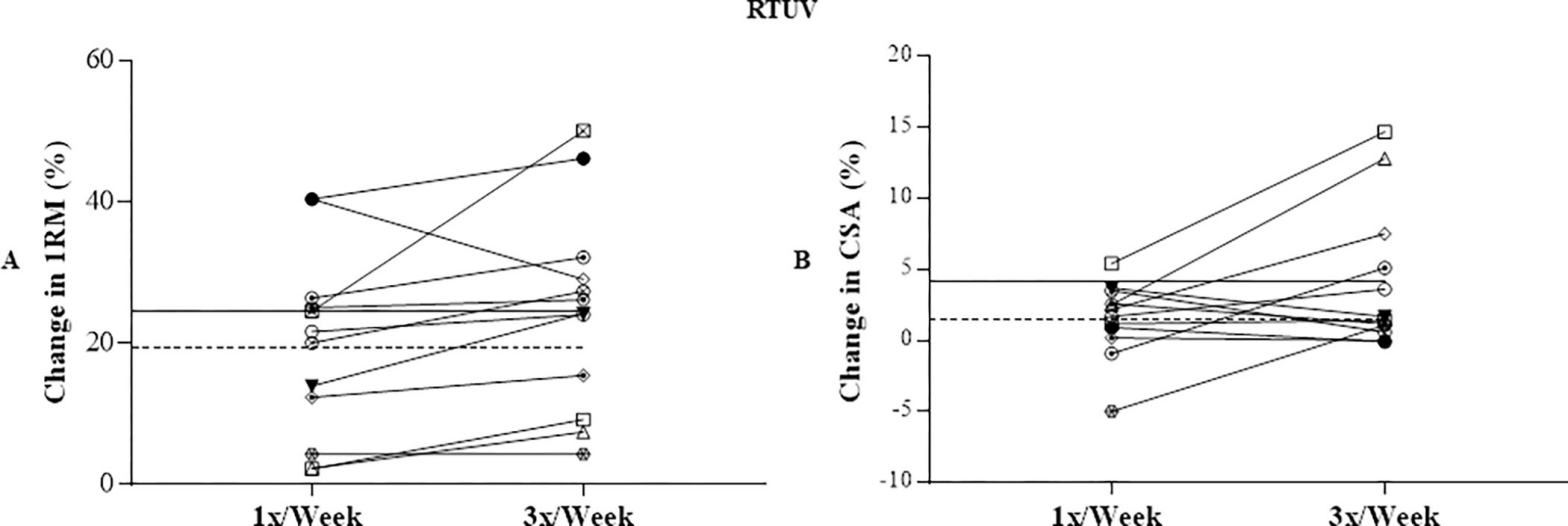
Individual percentage change in the values of 1RM (A) and CSA (B) in the RTUV. 1RM, maximum dynamic strength; CSA, quadriceps femoris cross-sectional area; RTUV, resistance training with unequalized total training volume; ———, average of the percentage change 1x/week;–––––, average of the percentage change 3x/week.

Considering the individual changes in the 1RM and CSA values (percentage changes), the mean of individual % changes between 1x and 3x were smaller in the **RTEV** condition (Fig 4A and 4B) compared to **RTUV** condition (Fig 5A and 5B).

## Discussion

The present study verified the changes in lower limbs 1RM performance and quadriceps femoris muscle CSA after a 9-week RT program executed at different weekly frequencies (one and three times) in a group of trained individuals with a within-subject design. Our main findings showed that there were no significant differences in 1RM and CSA values between the different RT frequencies, regardless of whether or not TTV was equalized. However, ES and CI suggested a greater training effect on 1RM and CSA induced by higher RT frequency compared with lower in the RTUV condition, where the lower limb that trained three times a week showed a higher TTV compared to the contralateral limb once/week. On the other hand, in the **RTEV** a small training effect was induced by 3x/week compared with 1x/week. In this condition, lower limbs TTV was equalized between different RT frequencies.

McLester Jr. et al. [35] were the first to compare different RT frequencies with equalized volumes in trained individuals. In that study, they compared RT protocols where each muscle group was trained one or three times/week, and significant increases in leg press 1RM values were observed in the group with higher weekly frequency (22.3% *vs*. 46.1% for one and three times/week, respectively). The divergence found between these results and ours may be explained by two important facts. 1) McLester Jr. et al. [35] applied a between-subject design, which favors higher data variability in RT-induced changes in strength gains [28] which might, at least partially, explain the differences between intervention groups; and 2) the initial diferences in 1RM values presented by the participants of both studies. In the study of McLester Jr. et al. [35] the baseline values of 1RM in the bilateral leg press were 200.3 ± 83.1 kg and 191.2 ± 96.3 kg, for the frequencies of one and three times/week, respectively. In our study, these values were higher (Table 2) considering that the unilateral leg press 45° was the applied test. Apparently, there is a negative relationship between the initial strength level and the percentage of its increase during the training period [36]. According to the American College of Sports Medicine [7], untrained individuals can increase muscle strength by approximately 40% over training periods ranging from four weeks to two years. In contrast, in trained individuals these increases may be approximately 16% for the same period, closer to the results found in this study.

Also comparing weekly RT frequencies of one or three times/week in trained individuals, Schoenfeld et al. [22] did not found significant differences in 1RM increases in the bench press (6.8% and 10.2%) and squat (10.6% and 11.3%) exercises, for the RT performed one and three times a week, respectively. Indeed, the literature has not shown differences in muscle strength gains when different RT frequencies were compared in trained individuals, regardless of whether the TTV was equalized [22, 37–41] or not [42–45]. However, these studies also have not considered between-subjects’ data variability in their design. An important feature of the present study was the use of a within-subject design to decrease this variability, also allowing a rigorous equalization of the TTV performed weekly. Moreover, this is the first study to bring in the same experimental design the comparison of different RT frequencies with equalized (**RTEV**) and unequalized (**RTUV**) TTV.

From a more practical approach, in the present study, **RTEV** and **RTUV** conditions allowed to us making within- and between-subject comparisons. Considering the within-subject comparison, in the **RTEV** condition (*i*.*e*., different RT frequencies with same TTV), the training effect on strength performance was, in fact, similar between low and high RT frequencies. However, in the **RTUV** condition (*i*.*e*., different RT frequencies and TTV), the training effect on strength gain was more pronounced in the high RT frequency and high TTV (supported by the use of the ES and CI). It is noteworthy that in between-subject approach, the comparison between high RT frequencies (*i*.*e*., 3x/week) in the **RTEV** and **RTUV** conditions, showed that the **RTUV** condition presented better results on strength performance (S1 Fig). Considering the same TTV between the different conditions (**RTEV** and **RTUV**), one could expect the same training-induced adaptation in muscle strength performance. Even though this result would be hard to reconcile, the between-subjects data variability seems to be a reasonable explanation for the observed difference [28]. Thus, it is possible to suggest that the between-subject approach may be less appropriate for investigating the effects of RT frequency and volume on muscular adaptations.

Differently from the McLester Jr. et al. [35], that estimated muscle mass increase and body composition alterations through skinfold measurements, most recent studies performed these same measurements through dual-energy x-ray absorptiometry [38, 39, 42, 45], air displacement plethysmography [46], and ultrasound images [22, 37, 39–41, 43, 44, 46]. In many of these studies [22, 39–41, 44, 46] authors did not find differences in lower limbs muscle CSA when different RT frequencies were compared. However, Zaroni et al. [43] demonstrated that a greater TTV provided by the higher RT frequencies could contribute to the increase in lower limb muscle CSA. As far as we know, this is the first study with trained individuals that used magnetic resonance imaging to assess changes in muscle CSA following a protocol with different weekly RT frequencies. In the present study no significant differences were observed in increases of quadriceps femoris CSA between the different frequencies of the **RTEV** condition. In fact, studies with trained individuals have not shown additional benefits to hypertrophic adaptations when the same TTV is distributed with different strategies [47, 48]. In the **RTUV** condition, no significant differences in CSA increases were also observed between the different RT frequencies; however, the ES and CI suggest a great effect on CSA induced by the higher RT frequency, probably due to the higher TTV (*i*.*e*., ES = 0.63; CI = 0.21 to 1.10). The ES interpretation corroborates the recent evidence demonstrating the importance of training volume in muscle adaptations [9, 10]. In practice, increasing the RT frequency would be one of the possible ways to increase TTV over a specific period of time [9, 15, 42–45].

Finally, as similarly discussed for the strength gains, the comparisons between high training frequency in the **RTEV** and **RTUV** conditions (*i*.*e*., between-subjects’ comparisons) showed that **RTUV** presented greater muscle CSA increments (see S1 Fig). In both conditions, the subjects trained three times per week and the TTV was similar, so similar adaptations would be expected for both groups, as observed in within-subject comparisons. The reasonable explanation for data discrepancy, strengthened by our experimental design, is also that between-subject data variability is imperative to interfere in the results, likely adding bias to the results.

It is important to mention some limitations of the present study: (a) CSA was measured only in the middle portion of the quadriceps femoris muscle (*i*.*e*., 50% of the distance between the greater trochanter and the lateral femoral epicondyle), precluding any speculation about hypertrophic adaptations in the proximal and distal regions; (b) only one exercise (unilateral leg press 45°) was performed during the 9-week RT program and it has been shown that varying exercises may result in larger hypertrophy adaptations [49]; (c) the unilateral RT protocol may promote a cross-education effect, which would increase contralateral limb strength gains [50]; however, this effect is less likely to occur in trained individuals that perform unilateral RT in both lower limbs [47]. In addition, the within-subject experimental design proposed in this study attempted to reduce the interference of the variability found in the between-subject experimental designs; (d) in the **RTEV** condition, the repetitions performed in the higher RT frequency could not always be considered maximum repetitions, as they were performed with the average weight obtained with the lower weekly frequency of the contralateral lower limb. However, recent evidence has shown that trained individuals in RT programs with equalized TTV can achieve similar or even higher results in both strength [51, 52] and muscle hypertrophy [53], even when maximum repetitions are not performed.

## Conclusion

The results of this study demonstrated that RT performed three times a week, increased 1RM and quadriceps femoris CSA similarly to that performed only once a week in trained subjects. When the higher RT frequency resulted in a larger TTV, such as in the **RTUV** condition (17% more than the lower frequency), greater ES were observed for both 1RM and CSA. Therefore, if trained individuals need higher RT volumes to obtain gains in both strength [8] and muscle mass [9–11], alternatives such as increasing RT frequency could be considered [21, 54]. Moreover, when the same TTV is distributed at different weekly frequencies, no additional benefits in increases in strength and muscle mass are observed.

## Supporting information

S1 Fig Effect size with the confidence interval in the highest resistance training frequencies (*i*.*e*., three times a week) in inter-subject experimental design comparisons. 1RM = 0.44 (ES) with 0.02 to 0.89 (CI) and, CSA = 0.48 (ES) with 0.05 to 0.94 (CI). ES, effect size; 1RM, maximum dynamic strength; CSA, quadriceps femoris cross-sectional area; CI, confidence interval.
(TIF)

## References

[1] Hass CJ, Feigenbaum MS, Franklin BA. Prescription of resistance training for healthy populations. Sports Med. 2001; 31: 953–964. doi: 10.2165/00007256-200131140-00001 11735680

[2] Ralston GW, Kilgore L, Wyatt FB, Buchan D, Baker JS. Weekly training frequency effects on strength gain: a meta-analysis. Sports Med. Open 2018; 4: 36. doi: 10.1186/s40798-018-0149-9 30076500 PMC6081873

[3] Kraemer WJ, Ratamess NA. Fundamentals of resistance training: progression and exercise prescription. Med Sci Sports Exerc. 2004; 36: 674–688. doi: 10.1249/01.mss.0000121945.36635.61 15064596

[4] American College of Sports Medicine. American College of Sports Medicine position stand. Progression models in resistance training for healthy adults. Med Sci Sports Exerc. 2009; 41: 687–708. doi: 10.1249/MSS.0b013e3181915670 19204579

[5] Schoenfeld BJ, Ogborn D, Krieger JW. Effects of resistance training frequency on measures of muscle hypertrophy: a systematic review and meta-analysis. Sports Med. 2016; 46: 1689–1697. doi: 10.1007/s40279-016-0543-8 27102172

[6] Fisher J, Steele J, Low SB, Smith D. Evidence-based resistance training recommendations. Med Sport. 2011; 15: 147–162. doi: 10.2478/v10036-011-0025-x

[7] American College of Sports Medicine. American College of Sports Medicine position stand. Progression models in resistance training for healthy adults. Med Sci Sports Exerc. 2002; 34: 364–380. doi: 10.1097/00005768-200202000-00027 11828249

[8] Ralston GW, Kilgore L, Wyatt FB, Baker JS. The effect of weekly set volume on strength gain: a meta-analysis. Sports Med. 2017; 47: 2585–2601. doi: 10.1007/s40279-017-0762-7 28755103 PMC5684266

[9] Figueiredo VC, de Salles BF, Trajano GS. Volume for muscle hypertrophy and health outcomes: the most effective variable in resistance training. Sports Med. 2018; 48: 499–505. doi: 10.1007/s40279-017-0793-0 29022275

[10] Schoenfeld BJ, Ogborn D, Krieger JW. Dose-response relationship between weekly resistance training volume and increases in muscle mass: a systematic review and meta-analysis. J Sports Sci. 2017; 35: 1073–1082. doi: 10.1080/02640414.2016.1210197 27433992

[11] Schoenfeld BJ, Contreras B, Krieger J, Grgic J, Delcastillo K, Belliard R, et al. Resistance training volume enhances muscle hypertrophy but not strength in trained men. Med Sci Sports Exerc. 2019; 51: 94–103. doi: 10.1249/MSS.0000000000001764 30153194 PMC6303131

[12] Ochi E, Maruo M, Tsuchiya Y, Ishii N, Miura K, Sasaki K. Higher training frequency is important for gaining muscular strength under volume-matched training. Front Physiol. 2018; 9: 744. doi: 10.3389/fphys.2018.00744 30013480 PMC6036131

[13] Johnsen E, Tillaar R. Effects of training frequency on muscular strength for trained men under volume matched conditions. PeerJ. 2021; 9: e10781. doi: 10.7717/peerj.10781 33643706 PMC7897409

[14] Blanco FP, Rosell DR, Aagaard P, Medina LS, Serna JR, Custodio RM, et al. Time course of recovery from resistance exercise with different set configurations. J Strength Cond Res. 2020; 34: 2867–2876. doi: 10.1519/JSC.0000000000002756 30036284

[15] Häkkinen K, Kallinen M. Distribution of strength training volume into one or two daily sessions and neuromuscular adaptations in female athletes. Electromyogr Clin Neurophysiol. 1994; 34: 117–124. 8187678

[16] Hartman MJ, Clark B, Bembens DA, Kilgore JL, Bemben MG. Comparisons between twice-daily and once-daily training sessions in male weight lifters. Int J Sports Physiol Perform. 2007; 2: 159–169. doi: 10.1123/ijspp.2.2.159 19124903

[17] Raastad T, Kirketeig A, Wolf D, Paulsen G. Powerlifters improved strength and muscular adaptations to a greater extent when equal total training volume was divided into 6 compared to 3 training sessions per week (abstract). Book of abstracts, 17th annual conference of the European College of Sport Science; 2012.

[18] Williams TD, Tolusso DV, Fedewa MV, Esco MR. Comparison of periodized and non-periodized resistance training on maximal strength: a meta-analysis. Sports Med. 2017; 47: 2083–2100. doi: 10.1007/s40279-017-0734-y 28497285

[19] Damas F, Phillips S, Vechin FC, Ugrinowitsch C. A review of resistance training-induced changes in skeletal muscle protein synthesis and their contribution to hypertrophy. Sports Med. 2015; 45: 801–807. doi: 10.1007/s40279-015-0320-0 25739559

[20] Dankel SJ, Mattocks KT, Jessee MB, Buckner SL, Mouser JG, Counts BR, et al. Frequency: the overlooked resistance training variable for inducing muscle hypertrophy? Sports Med. 2017; 47: 799–805. doi: 10.1007/s40279-016-0640-8 27752983

[21] Damas F, Phillips SM, Libardi CA, Vechin FC, Lixandrão ME, Jannig PR, et al. Resistance training-induced changes in integrated myofibrillar protein synthesis are related to hypertrophy only after attenuation of muscle damage. J Physiol. 2016; 594: 5209–5222. doi: 10.1113/JP272472 27219125 PMC5023708

[22] Schoenfeld BJ, Ratamess NA, Peterson MD, Contreras B, Tiryaki-Sonmez G. Influence of resistance training frequency on muscular adaptations in well-trained men. J Strength Cond Res. 2015; 29: 1821–1829. doi: 10.1519/JSC.0000000000000970 25932981

[23] Hammarström D, Øfsteng S, Koll L, Hanestadhaugen M, Hollan I, Apró W, et al. Benefits of higher resistance-training volume are related to ribosome biogenesis. J Physiol. 2020; 598: 543–565. doi: 10.1113/JP278455 31813190

[24] Brook MS, Wilkinson DJ, Smith K, Atherton PJ. It’s not just about protein turnover: the role of ribosomal biogenesis and satellite cells in the regulation of skeletal muscle hypertrophy. Eur J Sport Sci. 2019; 19: 952–963. doi: 10.1080/17461391.2019.1569726 30741116

[25] Grgic J, Schoenfeld BJ, Davies TB, Lazinica B, Krieger JW, Pedisic Z. Effect of resistance training frequency on gains in muscular strength: a systematic review and meta-analysis. Sports Med. 2018; 48: 1207–1220. doi: 10.1007/s40279-018-0872-x 29470825

[26] Grgic J, Schoenfeld BJ, Latella C. Resistance training frequency and skeletal muscle hypertrophy: a review of available evidence. J Sci Med Sport. 2019; 22:361–370. doi: 10.1016/j.jsams.2018.09.223 30236847

[27] Schoenfeld BJ, Grgic J, Krieger J. How many times per week should a muscle be trained to maximize muscle hypertrophy? A systematic review and meta-analysis of studies examining the effects of resistance training frequency. J Sports Sci. 2019; 37: 1286–1295. doi: 10.1080/02640414.2018.1555906 30558493

[28] Damas F, Barcelos C, Nóbrega SR, Ugrinowitsch C, Lixandrão ME, Santos LMED, et al. Individual muscle hypertrophy and strength responses to high vs. low resistance training frequencies. J Strength Cond Res. 2019; 33: 897–901. doi: 10.1519/JSC.0000000000002864 30289872

[29] Schoenfeld BJ, Grgic J. Evidence-based guidelines for resistance training volume to maximize muscle hypertrophy. J Strength Cond Res. 2018; 40: 107–112. doi: 10.1519/SSC.0000000000000363

[30] Brown LE, Weir JP. ASEP procedures recommendation I: accurate assessment of muscular strength and power. JEPonline 2001; 4: 1–21.

[31] Weir JP. Quantifying test-retest reliability using the intraclass correlation coefficient and the SEM. J Strength Cond Res. 2005; 19: 231–240. doi: 10.1519/15184.1 15705040

[32] Espiríto-Santo H, Daniel F. Calculating and reporting effect sizes on scientific papers: p < 0.05 limitations in the analysis of mean differences of two groups. Port J Behav Soc Res. 2015; 1:3–16.

[33] Nakagawa S, Cuthill IC. Effect size, confidence interval and statistical significance: a practical guide for biologists. Biol Rev Camb Philos Soc. 2007; 82: 591–605. doi: 10.1111/j.1469-185X.2007.00027.x 17944619

[34] Cohen J. Statistical power analysis for the behavioral sciences. Hillsdale: Lawrence Erlbaum Associates; 1988.

[35] McLester JR, Bishop P, Guilliams M. Comparison of 1 day and 3 days per week of equal-volume resistance training in experienced subjects. J Strength Cond Res. 2000; 14:273–281.

[36] Häkkinen K. Factors influencing trainability of muscular strength during short and prolonged training. Natl Strength Cond Assoc J. 1985; 7:32–37.

[37] Colquhoun RJ, Gai CM, Aguilar D, Bove D, Dolan J, Vargas A, et al. Training volume, not frequency, indicative of maximal strength adaptations to resistance training. J Strength Cond Res. 2018; 32: 1207–1213 doi: 10.1519/JSC.0000000000002414 29324578

[38] Fortes LS, Costa MC, Ferreira M, Nascimento-Júnior J, Fiorese L, Lima-Júnior D, et al. Frequency of resistance training does not affect inhibitory control or improve strength in well-trained young adults. *PLoS One* 2018; 13: e0206784. doi: 10.1371/journal.pone.0206784 30388181 PMC6214533

[39] Hamarsland H, Moen H, Skaar OJ, Jorang PW, Rødahl HS, Rønnestad BR. Equal-volume strength training with different training frequencies induces similar muscle hypertrophy and strength improvement in trained participants. Front Physiol. 2022; 12: 789403. doi: 10.3389/fphys.2021.789403 35069251 PMC8766679

[40] Lasevicius T, Schoenfeld BJ, Grgic J, Laurentino G, Tavares LD, Tricoli V. Similar muscular adaptations in resistance training performed two versus three days per week. J Hum Kinet. 2019; 68: 135–143. doi: 10.2478/hukin-2019-0062 31531139 PMC6724585

[41] Saric J, Lisica D, Orlic I, Grgic J, Krieger JW, Vuk S, et al. Resistance training frequencies of 3 and 6 times per week produce similar muscular adaptations in resistance-trained men. J Strength Cond Res. 2019; 33 (Suppl1), S122–S129. doi: 10.1519/JSC.0000000000002909 30363041

[42] Thomas MH, Burns SP. Increasing lean mass and strength: a comparison of high frequency strength training to lower frequency strength training. Int J Exerc Sci. 2016; 9: 159–167. 27182422 10.70252/HDLQ5133 PMC4836564

[43] Zaroni RS, Brigatto FA, Schoenfeld BJ, Braz TV, Benvenutti JC, Germano MD, et al. High resistance-training frequency enhances muscle thickness in resistance-trained men. J Strength Cond Res. 2019; 33 (Suppl 1): S140–S151. doi: 10.1519/JSC.0000000000002643 31260419

[44] Brigatto FA, Braz TV, Zanini TCDC, Germano MD, Aoki MS, Schoenfeld BJ, et al. Effect of resistance training frequency on neuromuscular performance and muscle morphology after 8 weeks in trained men. J Strength Cond Res. 2019; 33: 2104–2116. doi: 10.1519/JSC.0000000000002563 29528962

[45] Gomes GK, Franco CM, Nunes PRP, Orsatti FL. High-frequency resistance training is not more effective than low-frequency resistance training in increasing muscle mass and strength in well-trained men. J Strength Cond Res. 2019; 33 (Suppl1), S130–S139. doi: 10.1519/JSC.0000000000002559 29489727

[46] Yue FL, Karsten B, Larumbe-Zabala E, Seijo M, Naclerio F. Comparison of 2 weekly-equalized volume resistance-training routines using different frequencies on body composition and performance in trained males. Appl Physiol Nutr Metab. 2018; 43: 475–481. doi: 10.1139/apnm-2017-0575 29216446

[47] Angleri V, Ugrinowitsch C, Libardi CA. Crescent pyramid and drop-set systems do not promote greater strength gains, muscle hypertrophy, and changes on muscle architecture compared with traditional resistance training in well-trained men. Eur J Appl Physiol. 2017; 117: 359–369. doi: 10.1007/s00421-016-3529-1 28130627

[48] Oliver JM, Jagim AR, Sanchez AC, Mardock MA, Kelly KA, Meredith HJ, et al. Greater gains in strength and power with intraset rest intervals in hypertrophic training. J Strength Cond Res. 2013; 27: 3116–3131. doi: 10.1519/JSC.0b013e3182891672 23736782

[49] Fonseca RM, Roschel H, Tricoli V, de Souza EO, Wilson JM, Laurentino GC, et al. Changes in exercises are more effective than in loading schemes to improve muscle strength. J Strength Cond Res. 2014; 28: 3085–3092. doi: 10.1519/JSC.0000000000000539 24832974

[50] Manca A, Dragone D, Dvir Z, Deriu F. Cross-education of muscular strength following unilateral resistance training: a meta-analysis. Eur J Appl Physiol. 2017; 117:2335–2354. doi: 10.1007/s00421-017-3720-z 28936703

[51] Carroll KM, Bernards JR, Bazyler CD, Taber CB, Stuart CA, DeWeese BH, et al. Divergent performance outcomes following resistance training using repetition maximums or relative intensity. Int J Sports Physiol Perform. 2018; 29: 1–28. doi: 10.1123/ijspp.2018-0045 29809061

[52] Vieira JG, Dias MRC, Lacio M, Schimitz G, Nascimento G, Panza P, et al. Resistance training with repetition to failure or not on muscle strength and perceptual responses. JEPonline 2019; 22: 165–175.

[53] Carroll KM, Bazyler CD, Bernards JR, Taber CB, Stuart CA, DeWeese BH, et al. Skeletal muscle fiber adaptations following resistance training using repetition maximums or relative intensity. Sports (Basel). 2019; 7: 169. doi: 10.3390/sports7070169 31373325 PMC6680702

[54] Schoenfeld B, Fisher J, Grgic J, Haun C, Helms E, Phillips S, et al. Resistance training recommendations to maximize muscle hypertrophy in an athletic population: position stand of the IUSCA. Int J Strength Cond. 2021; 1: 1–30. doi: 10.47206/ijsc.v1i1.81

