# Supplementary figures and images for: Effect of different training frequencies on maximal strength performance and muscle hypertrophy in trained individuals—a within-subject design
Source: PLoS One. 2022 Oct 13;17(10):e0276154. doi: 10.1371/journal.pone.0276154 (PMC9560172; doi:10.1371/journal.pone.0276154)

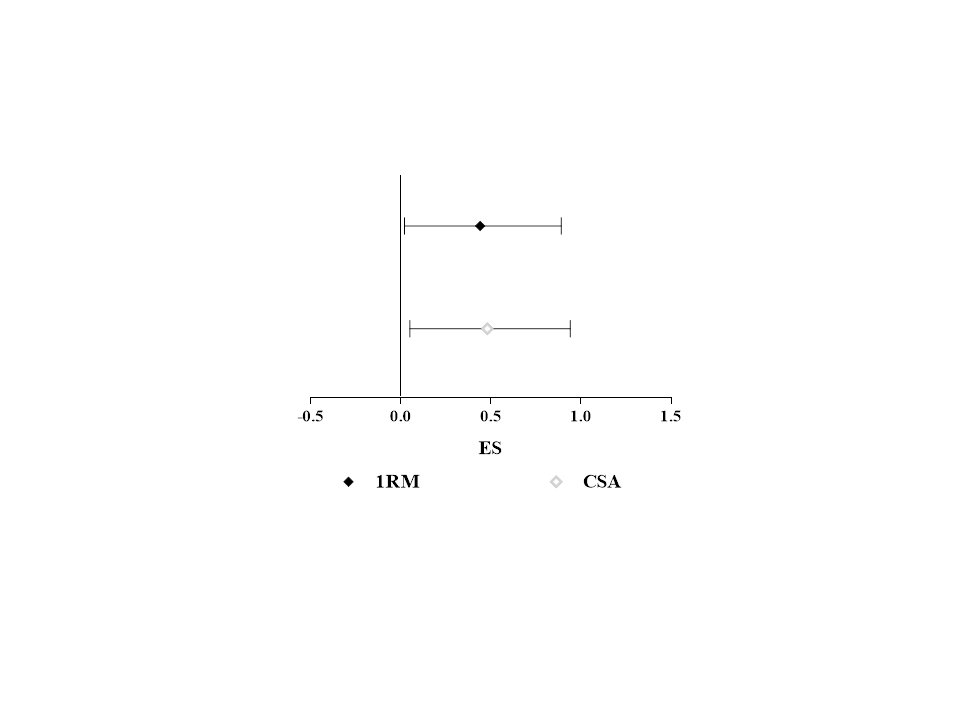

Supplement: S1 Fig — 1RM = 0.44 (ES) with 0.02 to 0.89 (CI) and, CSA = 0.48 (ES) with 0.05 to 0.94 (CI). ES, effect size; 1RM, maximum dynamic strength; CSA, quadriceps femoris cross-sectional area; CI, confidence interval. (TIF) [file pone.0276154.s001.TIF]
